# Objective Dual-Task Turning Measures for Return-to-Duty Assessment After Mild Traumatic Brain Injury: The ReTURN Study Protocol

**DOI:** 10.3389/fneur.2020.544812

**Published:** 2021-01-15

**Authors:** Peter C. Fino, Margaret M. Weightman, Leland E. Dibble, Mark E. Lester, Carrie W. Hoppes, Lucy Parrington, Jorge Arango, Alicia Souvignier, Holly Roberts, Laurie A. King

**Affiliations:** ^1^Department of Health and Kinesiology, University of Utah, Salt Lake City, UT, United States; ^2^Courage Kenny Research Center, Allina Health, Minneapolis, MN, United States; ^3^Department of Physical Therapy & Athletic Training, University of Utah, Salt Lake City, UT, United States; ^4^Army-Baylor University Doctoral Program in Physical Therapy, Fort Sam Houston, TX, United States; ^5^Department of Physical Therapy, Texas State University, Round Rock, TX, United States; ^6^Department of Neurology, Oregon Health & Science University, Portland, OR, United States; ^7^Traumatic Brain Injury Center of Excellence, Fort Carson, CO, United States; ^8^Evans Army Community Hospital, Fort Carson, CO, United States; ^9^Madigan Army Medical Center, Joint Base Lewis-McChord, WA, United States

**Keywords:** concussion, gait, return to sport (RTS), inertial sensors, wearable sensors

## Abstract

Determining readiness for duty after mild traumatic brain injury (mTBI) is essential for the safety of service members and their unit. Currently, these decisions are primarily based on self-reported symptoms, objective measures that assess a single system, or standardized physical or cognitive tests that may be insensitive or lack ecological validity for warrior tasks. While significant technological advancements have been made in a variety of assessments of these individual systems, assessments of isolated tasks are neither diagnostically accurate nor representative of the demands imposed by daily life and military activities. Emerging evidence suggests that complex tasks, such as dual-task paradigms or turning, have utility in probing functional deficits after mTBI. Objective measures from turning tasks in single- or dual-task conditions, therefore, may be highly valuable for clinical assessments and return-to-duty decisions after mTBI. The goals of this study are to assess the diagnostic accuracy, predictive capacity, and responsiveness to rehabilitation of objective, dual-task turning measures within an mTBI population. These goals will be accomplished over two phases. Phase 1 will enroll civilians at three sites and active-duty service members at one site to examine the diagnostic accuracy and predictive capacity of dual-task turning outcomes. Phase 1 participants will complete a series of turning tasks while wearing inertial sensors and a battery of clinical questionnaires, neurocognitive testing, and standard clinical assessments of function. Phase 2 will enroll active-duty service members referred for rehabilitation from two military medical treatment facilities to investigate the responsiveness to rehabilitation of objective dual-task turning measures. Phase 2 participants will complete two assessments of turning while wearing inertial sensors: a baseline assessment prior to the first rehabilitation session and a post-rehabilitation assessment after the physical therapist determines the participant has completed his/her rehabilitation course. A variable selection procedure will then be implemented to determine the best task and outcome measure for return-to-duty decisions based on diagnostic accuracy, predictive capacity, and responsiveness to rehabilitation. Overall, the results of this study will provide guidance and potential new tools for clinical decisions in individuals with mTBI.

**Clinical Trial Registration**: clinicaltrials.gov, Identifier NCT03892291.

## Introduction

Determining readiness for duty after mild traumatic brain injury (mTBI) is essential for the safety of service members (SMs) and their units. Sensory, motor, and cognitive deficits such as delayed reaction time (1), imbalance (2), and poorer cognitive functioning (3, 4) can pose serious risks to individuals following mTBI. Athletes with a recent mTBI are 60 to 70% more likely to experience a musculoskeletal injury over the subsequent 12 months compared to teammates without a recent mTBI, despite having no obvious clinical impairment, suggesting that these individuals are not fully ready for competitive activity when medically cleared after their mTBI (5–10). In SMs with mTBI, lasting impairments can have more severe implications. The ability to perform warrior tasks and battle drills, such as moving under fire while maintaining situational awareness, reacting to contact, and establishing security, is a critical component of combat effectiveness and survival for the SMs and their unit (11, 12). Additionally, the unit's mission readiness depends on the health of every SM; withholding an SM from duty requires convincing justification that they pose a tangible risk to themselves or their unit. Thus, return-to-duty (RTD) decisions should be made using objective assessments that are sensitive to mTBI-related deficits and are associated with functional performance in theater.

Currently, RTD decisions are based on self-reported symptoms, objective measures that assess a single system, or standardized physical tests that lack ecological validity for warrior tasks or battle drills. Duty-readiness is primarily assessed using patient-reported symptoms (13–16) and through batteries of clinical tests designed to detect single-system impairments in isolated sensory, balance, or cognitive domains. Decisions that hinge on self-reported symptoms do not assess function (12), and it is unclear whether self-reported symptoms are associated with job performance decrements that would constitute a tangible risk to SMs or their units. While persistent symptoms have been associated with lower quality of life and community integration (17–19), the impact of symptoms on comprehensive, functional performance is poorly understood (20, 21). Recently, the rehabilitation community has recognized the need to measure a multifaceted battery of assessments to characterize mTBI recovery (22–24), but these batteries remain a compilation of single-domain clinical assessments and may not be representative of the demands imposed by daily life and military activities (12, 25–29). The majority of warrior tasks (urban assault, building breaching, movement to contact, etc.) require the constant integration of complex sensory stimuli (visual, vestibular, and proprioceptive) while under cognitive load during movement in extreme and varying physical conditions (30, 31).

While understudied in mTBI populations (32), ambulatory turning is a complex task that requires the simultaneous integration of various systems that can be impaired after mTBI. Safe and effective turning is dependent on multiple systems and sophisticated integration between vestibular, ocular, somatosensory, motor, and cognitive functioning (33–35) to achieve coordinated head and body reorientation (36, 37). During turning, dynamic shifts in the body-sensed gravitoinertial acceleration reference frame (38) and asymmetrical loadings (39) alter vestibular and proprioceptive sensory information, requiring dynamic reweighting of sensorimotor information and sophisticated oculomotor reflexes to stabilize spatial information (38). Multisegmental coordination between the head, trunk, pelvis, and feet is required during turning to reorient the body and to account for the centripetal force and the increased risk of slips (40, 41) caused by transverse loads (39, 42–44). Even in a non-athlete population, turning is a critical functional movement; people generally turn 800 to 1,000 times per day (45), and more than 40% of daily steps are non-straight (46).

Turning velocity and head–body coordination during activities with cognitive demands are critical to an SM's ability to safely and effectively scan and maneuver through operational environments. The ability to successfully acquire, aim, and hit a target is a critical warrior task that requires rapid and coordinated reorientation of the head and body (47). Further, performance of a military agility drill during standard agility tasks is largely influenced by the quality of turning (48). Combining turning tasks with realistic cognitive demands may better reflect functional performance (12, 49–51). The ability to do two tasks at once is theorized to require executive control as attention must be appropriately allocated to successfully perform both tasks. Individuals with mTBI and more severe acquired brain injury show difficulty when performing simultaneous motor and cognitive tasks (52), including decreased walking speed, increased variability, decreased stability (53), and an impaired ability to perceive and avoid obstacles when walking (54). Warfighter tasks require decision-making and intact cognitive and sensory function and are often performed in dynamic and stressful environments requiring elite physical abilities. Simultaneous and dynamic measurement of turning in combination with appropriate and ecologically relevant cognitive overlay may be a sensitive and reliable paradigm to assess real-world function in a standardized and measurable way.

Quantifying turning performance has traditionally been difficult in clinical settings, but recent advances in wearable sensors have enabled objective, robust, reliable, and sensitive assessments of turning performance in other clinical populations (45, 55–59). This project is designed to transition our previous research findings (8, 9, 21, 45, 55–59) into clinical settings for improved assessments after mTBI by evaluating objective dual-task (DT) turning measures for use as rehabilitative outcomes and as tools for RTD assessments. Therefore, the first goal of this study is to assess diagnostic accuracy—the added value of objective DT turning measures over standard clinical assessments. We hypothesize (a) that objective turning measures, performed in DT contexts, will improve the diagnostic accuracy relative to standard clinical assessments of physical function among people with mTBI and (b) that objective turning measures, performed in DT contexts, will be associated with impairments in International Classification of Functioning, Disability, and Health (ICF) model components, including body functions and structures, activities, and participation domains (60). The second goal of this study is to determine predictive capacity—if objective DT turning measures predict functional performance in civilian and military relevant tasks. We hypothesize that objective turning measures, performed in DT contexts and obtained in the clinic, will predict functional performance in (a) ecologically relevant civilian environments and in (b) ecologically relevant simulated high-demand battle drills. Our third goal in this study is to assess responsiveness to rehabilitation—the responsiveness of objective DT turning measures to standard vestibular rehabilitation in active-duty SMs with residual mTBI-related symptoms. We hypothesize that objective turning measures, performed in DT contexts, will measurably improve over the course of customary rehabilitation for SMs with mTBI-related residual symptoms.

## Methods/Design

This study has two phases: for Phase 1, participants will be recruited from the general civilian populations surrounding three sites [Oregon Health & Science University (OHSU), University of Utah (UU), Courage Kenny Research Center (CKRC)] and active-duty SMs at one site [Fort Sam Houston (FSH)] to address the first two goals of the study (diagnostic accuracy and predictive capacity). For Phase 2, participants will be recruited from active-duty SMs referred to military medical treatment facilities (Evans Army Community Hospital and Madigan Army Medical Center) for physical therapy following mTBI to address the third goal of the study (responsiveness to rehabilitation). This study has been registered on ClinicalTrials.gov (NCT03892291).

### Phase 1: Diagnostic Accuracy and Predictive Capacity of Turning Measures

#### Participants

Phase 1 will include approximately 50 civilians with persistent symptoms from mTBI, 50 civilian healthy controls, and 40 active-duty SM healthy controls. Three non-military sites (OHSU, UU, CKRC) will test civilians. A fourth military site (FSH) will enroll and test 40 active-duty SM healthy controls across a range of military experience and ability levels.

#### Inclusion and Exclusion Criteria

Participants may be active duty (at FSH), veterans, or nonveterans and must (1) have a diagnosis of mTBI based on Veterans Affairs/Department of Defense (VA/DoD) criteria (61, 62), (2) be between 18 and 50 years old, and (3) be outside of the acute stage (>3 weeks postconcussion) according to the VA/DoD clinical practice guidelines (63) but within 3 years of their most recent mTBI and still reporting symptoms. Control participants must have no history of mTBI or be more than 7 years removed from their most recent mTBI and no reported residual symptoms. All participants must (1) not have had or currently have any other injury, medical, or neurological illness that could potentially explain balance deficits (e.g., central or peripheral nervous system disease, stroke, greater than mild TBI, lower extremity amputation, recent (<6 months) lower extremity or spine orthopedic injury requiring a profile); (2) meet criteria for moderate to severe substance-use disorder within the past month, as defined by *Diagnostic and Statistical Manual of Mental Disorders, Fifth Edition* (64); (3) display behavior that would significantly interfere with validity of data collection or safety during the study; (4) be in significant pain during the evaluation (7/10 by patient subjective report); (5) be a pregnant female (balance considerations); or (6) be unable to communicate in English.

#### Assessment Procedures

All assessment procedures for Phase 1 will occur in a single session. Potential participants will be screened for eligibility prior to scheduling their assessment and their arrival at the clinic. Upon arrival at the clinic, study team personnel will review the study procedures and obtain informed written consent from the participant. All participants will be assessed using a battery of assessments including self-reported questionnaires, computerized neurocognition, single-task and DT turning, ecologically relevant mobility tests, clinical mobility tests, and vestibular–ocular motor screening. All procedures have been approved by local institutional review boards and the US Army Medical Research and Materiel Command, Office of Research Protections, Human Research Protection Office.

#### Primary Outcome Measures—Diagnostic Accuracy

**Figure 1:**
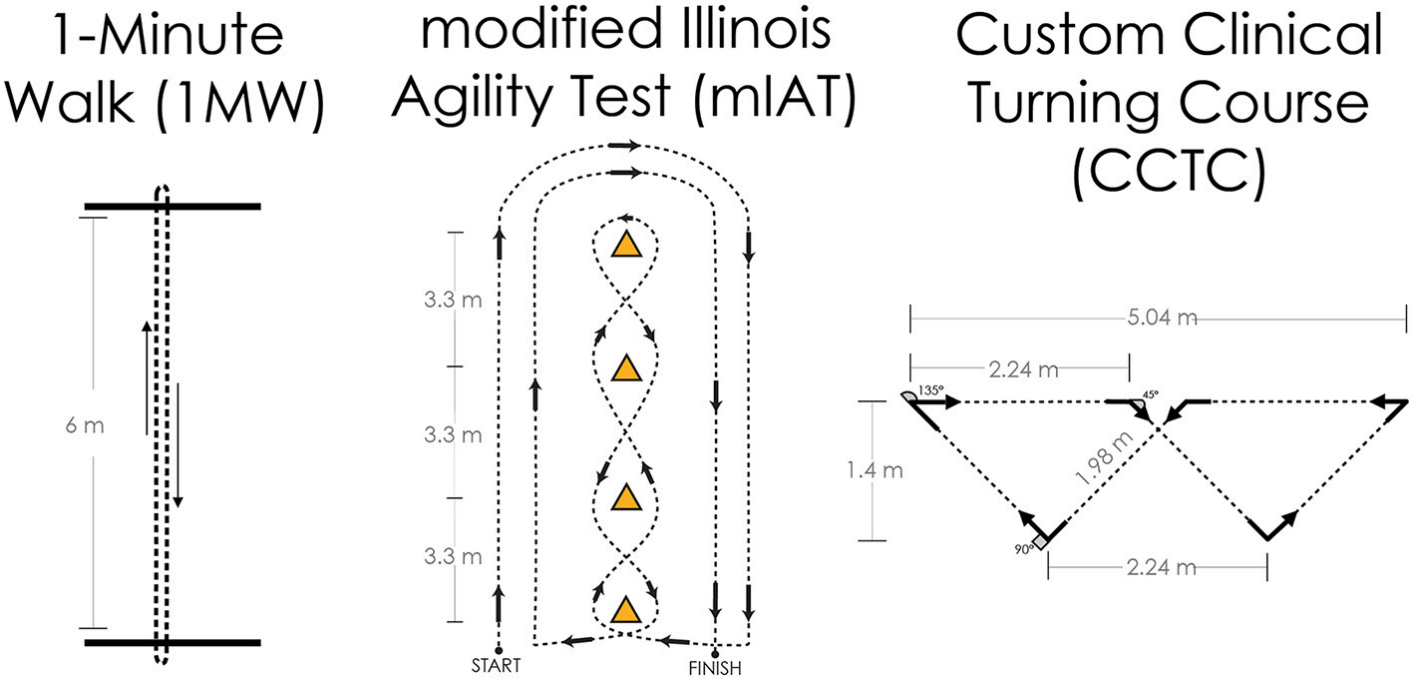
Overview of the three primary clinical turning tasks for this study protocol. Left: The 1-min walk (1MW) test is performed by walking at one's self-selected pace back and forth between two lines for 1 min; turns are assessed at the end of the walk, and all turns are 180°. Center: The modified Illinois Agility Test (mIAT) is a condensed version of the Illinois Agility Test and completed at one's maximum running speed; turns are assessed throughout the course and include 180° turns at the ends and shallower slalom turns in the middle. Right: The custom clinical turning course (CCTC) was designed to fit in a small hallway or large examination room and is completed at one's comfortable walking pace; turns are evaluated throughout the course and include 45°, 90°, and 135° turns representative of daily life.

The primary outcome measures will be obtained through a series of clinical turning tasks (Table 1). Each participant will complete three prescribed clinical turning assessments in the laboratory while being measured with wearable sensors. The prescribed tests will involve a 1-min walk test between two lines on the ground marked 6 m apart (1MW), a modified Illinois Agility Test (mIAT) (65, 66), and a custom clinical turning course (CCTC) designed to mimic turns performed in daily life (21) (Figure 1). All participants will perform one block of turning tasks. Each block will consist of one 1MW, one mIAT, and one CCTC performed with no additional cognitive task, and one 1MW, one mIAT, and one CCTC performed with a simultaneous cognitive overlay. The cognitive overlay for the 1MW and the mIAT involves an 8-digit grid coordinate memorization task. The overlay for the CCTC involves monitoring and responding to keywords in a custom-developed simulated radio chatter task (28). A subset of participants will complete a second block of turning tasks to establish test–retest reliability and minimum detectable change.

**Table 1:** Primary outcomes from objective turning tasks in phase 1.

	**Description**	**Purpose**
**Primary outcome**		
Peak lumbar turning speed (rad · s^−1^)^*^	Assesses the peak angular rate of the pelvis measured in the transverse plane	To assess whether the speed of reorientation changes after mTBI
**Other objective turning outcomes**		
Head turning speed (rad · s^−1^)^*^	Assesses the peak angular rate of the head measured in the transverse plane	To assess differences in the peak angular rate of superior body segments between people with mTBI and healthy controls
Upper trunk turning speed (rad · s^−1^)^*^	Assesses the peak angular rate of the upper trunk (sternum) measured in the transverse plane	
Head–body coordination timing (s)^*^	Assesses the time difference between peak angular velocity of the head and peak angular velocity of the trunk	To assess differences in head–body coordination during turning between people with mTBI and healthy controls
Peak head-pelvis angular displacement/RoM (°)	Assesses the peak angular displacement between the head and the trunk measured prior to or at the onset of trunk axial rotation when turning, measured in the transverse plane	
Head turn symmetry	Assesses the ratio of left-to-right head-turn velocity	
**Cognitive DT outcomes**		
Cognitive-task accuracy	Assesses the percentage of cognitive task responses that are correct in dual-task conditions	To compare whether cognitive performance when turning is impaired in people with mTBI
Number of correct responses per second	Assesses the overall number of correct responses, divided by the overall time in seconds	

* *These measures will involve assessment of both the mean and the variability (i.e., standard deviation)*.

#### Primary Outcome Measures—Predictive Capacity

To assess the capacity of DT turning measures to predict performance in a civilian ambulatory task (CAT), participants will navigate through an uncontrolled pedestrian environment while wearing inertial sensors as shown in Figure 1. A walking route of standardized length will be established at each site for participants to walk around the testing location. Common elements at each site's walking route will be written instructions and landmark-based directions to follow (e.g., take the second set of doors on your right). Each site's walking route will require participants to ambulate around public areas and will take ~7 min to complete. Participants will be required to navigate through crowded hallways, ascend and descend stairs, and scan for pedestrian traffic. A trained research assistant will accompany the participant throughout the entire route and provide verbal commands. The research assistant will remain behind the participant to not interfere with the navigation, but close enough to assist the participant in case of a loss of balance. Secondary landmarks will be known by the research assistants. If the participant misses a landmark, the research assistant will notify the participant once he/she reaches the secondary landmark, and the research assistant will instruct the participant that he/she traveled the wrong way and reread the instruction. The route will end in the initial testing area. The primary outcome of the CAT will be the time required to complete the route. Secondary outcome measures will include the peak head and trunk turning velocity, the head–body coordination throughout the course, and the number of landmarks identified and missed.

**Figure 2:**
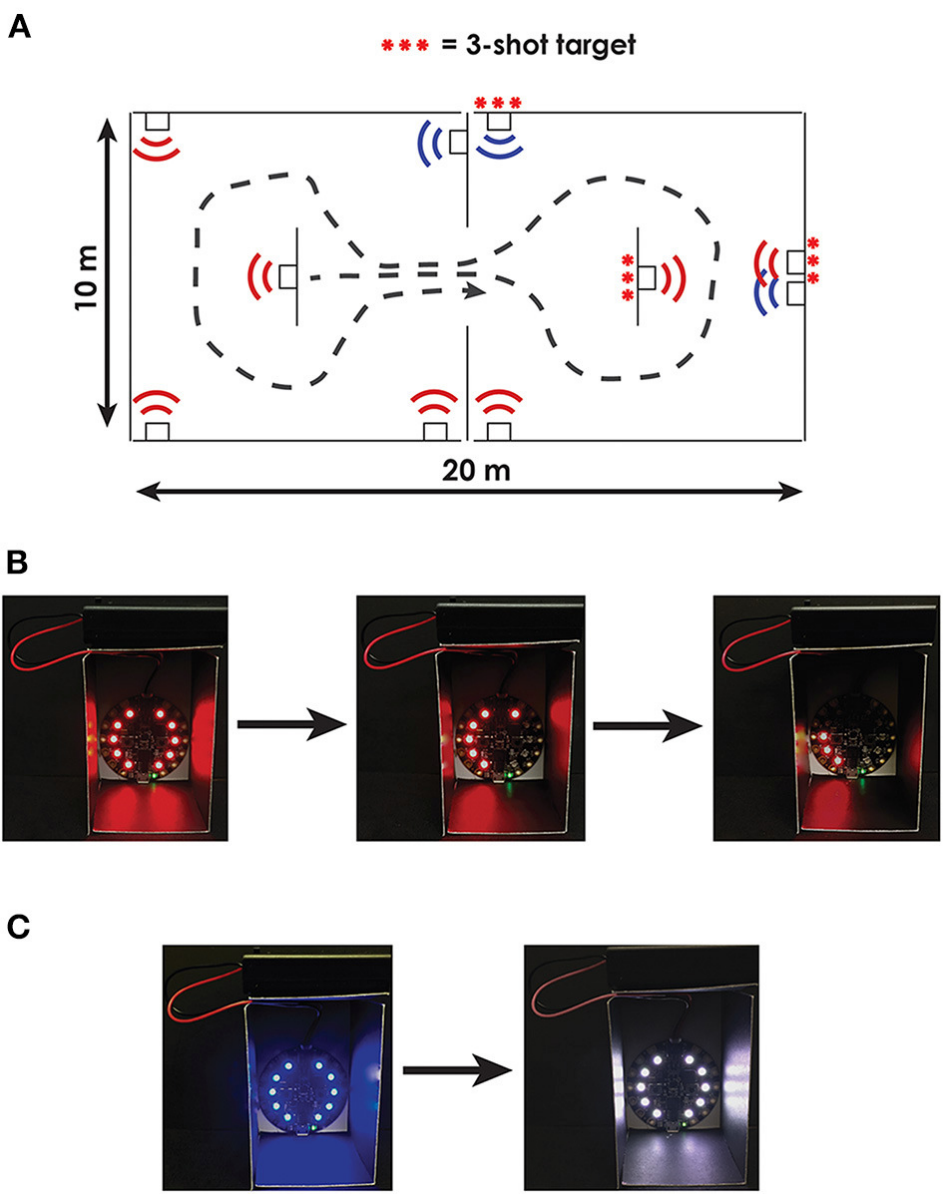
Overview of the Simulated Urban Patrol (SUP) task. **(A)** Bird's-eye view of the compartmentalized rooms including one configuration of hostile (red) and friendly (blue) targets. Some targets were configured to require multiple shots to clear; these targets are denoted with three red ***. **(B)** Example image of a hostile target that required three shots to clear. With each shot, 1/3 of the target's LEDs turn off; participants are instructed to completely clear all LEDs on the hostile targets. **(C)** Example image of a friendly target turning white when tagged indicating an incorrect shot.

To assess the capacity of DT turning measures to predict performance in a military-relevant task, participants will complete a simulated urban patrol task (SUP, Figure 2). During the SUP task, participants will navigate an environment containing two small rooms separated by a doorway. The environment will be created using portable partitioning boards to compartmentalize a medium-sized clinical or laboratory space. Each compartmentalized room within the environment will contain five 5-cm diameter targets, surrounded by LED lights. The targets will be placed in areas that are not visible from the adjoining compartment. The LED lights surrounding each target will denote either hostile (illuminated red) or friendly (illuminated blue) targets.

Participants will be instructed to enter the environment and secure the area as quickly as possible by clearing all hostile targets without harming friendly targets. Participants will use a mock weapon with a trigger-activated laser (i.e., laser gun) to clear targets. To clear hostile targets, participants will need to accurately scan each room and tag the hostile target with their laser. Each target will contain infrared-sensing diodes that will identify when the target was tagged with the laser. Upon tagging each target with a laser, the LEDs surrounding the hostile targets will turn off, while the LEDs surrounding the friendly targets will turn white (indicating an incorrect shot). Following the task, participants will also be asked to recount the number of hostile targets and number of friendly targets in each room. The primary outcome of functional performance for the simulated SUP task will be a throughput score calculated using the Comstock method (67), which allows unlimited rounds and penalizes shooting at the wrong target twice the score of shooting at the correct target (i.e., shooting a friendly target is twice as bad as missing a hostile target). Therefore, each trial will have a maximum number of possible accuracy points: Possible points = 2 * (Number of friendly targets) + 1 * (Number of hostile targets). The final accuracy score will be Accuracy score = Possible points – 2 * (Number of tagged friendly targets) – 1 * (Number of untagged hostile targets). The final throughput score will then be calculated by dividing the accuracy score by the time to complete the task: Throughput = Accuracy score/Completion time. Secondary outcomes will include objective measures of movement, including peak head and trunk turning velocity and head–body coordination, target accuracy, and the accuracy of the hostile / friendly target recall.

#### Sample Size

Sample size calculation and power analysis were based on Hedge's *g* effect sizes determined through preliminary data on turning in people with mTBI (21); we observed a range of Hedge's *g* effect sizes of −1.09 to −1.32 between the peak lumbar turn velocity of healthy controls and people with mTBI and residual symptoms when performing 90° turns. With 50 civilian participants per group and the midrange effect size from our preliminary work, we will have 90% power to detect differences in peak lumbar turning velocity between healthy controls and people with mTBI with a two-tailed 0.01 significance level if we observe at least 65% of the effect size we observed previously (*g* = −1.20). For the second goal, the power analysis was based on the correlation between clinic-based peak turning velocity and the duration of walking bouts, a common measure of daily ambulatory function, exhibited during daily, unmonitored activities at home (Pearson ρ = 0.362, data obtained from DoD #W81XWH-15-1-0620). With 140 planned participants (100 civilian participants and 40 active-duty participants), we will have 97% power to detect a correlation between the lumbar turning velocity measured in the clinic and daily function with a two-tailed 0.01 significance level if we observe the same effect size. Power calculations were performed in G^*^Power 3.1.9.2 (68).

#### Secondary Outcome Measures

Secondary outcome measures of symptomology, cognition, static balance, and clinical measures of mobility are provided in Table 2. Participation level outcomes will be assessed using the Neurobehavioral Symptom Inventory (NSI) (69), Dizziness Handicap Inventory (DHI) (70), and Quality of Life after Brain Injury (QOLIBRI) questionnaires (71). Clinical assessments of activity level will include postural sway during static quiet standing, the Functional Gait Assessment (FGA) (72), and the High Level Mobility Assessment Tool (HiMAT) (73–75). Assessments of body function and structure will include the Vestibular Ocular Motor Screening (VOMS) (76) and the Automated Neuropsychological Assessments Metrics (ANAM) (77). In agreement with the Common Data Elements (CDE) recommendations, covariates aligning with the National Institute of Neurological Disorders and Stroke and Federal Interagency Traumatic Brain Injury Research CDEs will also be recorded. Covariates and confounders will include (1) significant medical history and mTBI history based on a modified version of the Ohio State University TBI Identification Method (prior mTBIs, loss or alteration of consciousness, length of posttraumatic amnesia) and (2) posttraumatic stress disorders (PTSD) using the PTSD Checklist for Civilians and PTSD Checklist for Veterans (78, 79). Military Occupational Specialty (for enlisted and warrant officers) or Area of Concentration (for commissioned officers) for Army participants, Air Force Specialty Code for Air Force participants, and Navy Enlisted Classification (for enlisted SMs) or Commissioned Officer Designator (for commissioned officers) for Navy participants will be collected to further characterize the active-duty military sample.

**Table 2:** Secondary outcome measures for phase 1.

**Outcome measures**	**Description**	**Purpose**
**Participation level, ICF**		
*Questionnaires*		To determine the criterion validity of objective measures of DT turning with perceived handicap, symptom severity, and quality of life
Neurobehavioral symptom inventory	Assesses self-perceived symptoms associated with mTBI	
Dizziness handicap inventory	Assesses self-perceived handicap imposed by dizziness	
Quality of life after brain injury	Assesses the effects of TBI on quality of life	
**Activities level, ICF**		
*Clinical assessments of activities*		To assess the value of objective measures of DT turning over current clinical assessments of activities
Static sway (ML-RMS of sway)	Assesses static postural stability	
Functional gait assessment (total score)	Assesses dynamic balance by measuring performance on simple and complex gait tasks	
High level mobility assessment tool (total score)	Assesses dynamic balance by measuring performance on rapid and challenging mobility tasks	
**Body functions and structures level, ICF**		
*Clinical assessments of body functions and structures*		
Vestibular ocular motor screening (VOMS)	Assesses ocular–motor function and the provocation of symptoms	To compare the diagnostic accuracy of objective measures of DT turning to current clinical assessments of body functions and structures
Automated neuropsychological assessment metrics (ANAM)	Assesses cognitive domains of attention, memory, reaction time, processing speed, and executive function	

#### Statistical Analysis

To evaluate the capability of objective, DT turning measures to discriminate between healthy controls and people with mTBI, we will construct receiver operating characteristic (ROC) curves using each objective turning outcome and clinical assessment of activity. The area under the ROC curve (AUC) will be calculated, and AUC confidence intervals will be generated through a bootstrapping procedure. We will compare the AUCs and confidence intervals between each objective turning measure and clinical assessment of activity using tests of equality and a 0.05 significance level with Holm-Bonferroni corrections to adjust for multiple comparisons.

To assess the capacity of DT turning measures to predict performance in civilian or military-relevant tasks, we will use Pearson correlation coefficients and linear regression models. Pearson correlation coefficients will compare the linear relationship between objective turning outcomes and the primary outcome of task completion time during the CAT and throughput during the SUP tasks within participants with mTBI, healthy control participants, and all participants together. To determine how changes in objective turning measures correspond to changes in functional performance, linear regression models will be built for each objective turning measure that was significantly associated with functional performance. The primary outcomes for the civilian and military-relevant tasks will be modeled as the dependent variable. To account for potential differences in performance between civilians and active-duty SMs, a group effect will be included as a covariate. Beta coefficients will be interpreted to identify the relative change in objective turning measures that are associated with changes equal in magnitude to 1 standard deviation of the healthy control participants' performance.

### Secondary Statistical Analyses

To evaluate if turning outcomes have added value over standard clinical assessments of physical function, base multivariate logistic regression models will be constructed for each clinical assessment of physical function (e.g., FGA, HiMAT). A “base + 1” logistic regression model will be built by adding the objective turning measure with the highest AUC as described above. ROC curves will be constructed for the base and “base + 1” models, and the AUC calculated and compared using likelihood ratio tests. If the AUC from the “base + 1” model is greater than the AUC of the base model, then the process will be repeated iteratively—the outcome measure with the next largest univariate AUC will be added to the “base + 1” logistic regression model to create a “base + *n*” model until the addition of outcomes does not significantly change the AUC.

To evaluate if objective turning measures will be associated with impairments in ICF model components, including body functions and structures, activities, and participation domains, Pearson and Spearman correlation coefficients will be implemented separately within healthy civilian control participants, healthy active-duty control participants, and civilians with mTBI. Linear regression models with ICF impairments (Table 2) as dependent outcomes and main effects of objective turning outcomes, group, and the group ^*^ turning outcome interaction will assess whether the association between objective turning outcomes and ICF function varies by group through the interaction term. A 0.05 significance level will be used with stepwise Holm-Bonferroni corrections to adjust for multiple comparisons.

To determine clinically viable measures of turning based on clinometric properties, we will calculate the repeatability and minimum detectable difference using a Bland-Altman framework on the subset of participants who complete two blocks of turning tasks (80, 81). Linear mixed models will be fit for each objective outcome measure and task combination, with site location included as a covariate. Random intercepts will be fit for each participant to control for repeated measurements. Repeatability and minimum detectable change will be assessed using the within-subject error term for each outcome measure. Minimum detectable change will be calculated as $1.96^{*}\sqrt{2}$ times the within-subject error of the civilian control, military control, and civilian mTBI groups separately, as well as all groups combined. Differences across sites will be assessed using the standard error of effect of site location. Intraclass correlation coefficients will assess the absolute agreement and consistency of the outcomes across difference testing blocks. Outcomes with ICCs >0.7 will be identified as having good agreement and consistency for clinical implementation. Bias and learning effects will be considered by directly investigating the mean and median difference between blocks using a Bland-Altman framework (80, 81).

To determine whether healthy active-duty SMs perform turning tasks differently than healthy civilians, we will fit linear regression models for each turning outcome with main effects of group (active-duty SMs vs. civilians) and adjusted for age, body mass index, and other potential covariates. Inferences will be made using a significance level of 0.05 and a Holm-Bonferroni adjustment for multiple comparisons. If we find outcomes significantly differ between active-duty populations and control populations, we can conclude that population-specific normative values are needed. Given the large sample size of active-duty SMs and controls, we will then separate active-duty and civilian control groups for future analyses using those outcomes with significant differences between active-duty and civilian control participants. If we fail to find differences between active-duty and civilian control groups, it suggests performance does not vary by service status, and normative means, standard deviations, and minimum detectable changes will be calculated by combining data from all healthy control participants. We will also compare the distributions of performance using interval estimators to determine the extent to which the range of performance overlaps between active-duty SMs and civilians.

### Phase 2: Responsiveness to Rehabilitation

#### Participants

Phase 2 will include 40 active-duty SMs with persistent symptoms from mTBI who are referred for physical therapy because of their symptoms. Participants will be recruited from two military medical centers specializing in the rehabilitation of active-duty personnel after mTBI, the Evans Army Community Hospital (EACH) at Fort Carson, CO; and Madigan Army Medical Center (MAMC) at Joint Base Lewis-McChord, WA. Each site will recruit 20 participants for Phase 2. Participants will be tested before and after their rehabilitation; this study will have no influence or input on the rehabilitation prescribed or delivered to the participants.

#### Inclusion and Exclusion Criteria

All participants must be active-duty SMs who are referred to either the EACH or MAMC for physical therapy following mTBI. All other inclusion and exclusion criteria will be identical to those in Phase 1.

#### Assessment Procedures

Participants will complete two assessments: a baseline assessment prior to the first rehabilitation session and a postrehabilitation assessment after the physical therapist determines the participant has completed his/her rehabilitation course. At each assessment, patients will complete symptom inventories (NSI, DHI, QOLIBRI) and will be outfitted with inertial sensors. Participants will then complete the three turning tasks described in Phase I while wearing the sensors. One block will be performed, consisting of one 1MW, one mIAT, and one CCTC performed with no additional cognitive task, and one 1MW, one mIAT, and one CCTC performed while simultaneously performing a cognitively demanding task. At the postrehabilitation assessment, participants will complete the Patients' Global Impression of Change Scale (PGIC) (82). Participants' adherence to their rehabilitation program will be measured by their attendance at scheduled physical therapy visits.

#### Primary Outcome Measures—Responsiveness to Rehabilitation

To determine the responsiveness to rehabilitation, we will (1) determine the clinically important difference for objective turning outcomes and (2) compare the standardized response mean of each to rehabilitation to the minimum detectable change calculated in Phase I.

To determine the clinically important difference of turning outcomes, changes in objective turning outcomes will be anchored to the PGIC. Linear regression models will be fit to determine the differences in objective turning outcomes that are associated with a unit improvement in the patients' activity, symptoms, emotions, and overall quality of life, based on the PGIC.

To compare the effect of rehabilitation to the minimum detectable change, calculated from Phase 1, for each outcome, we will compute the mean difference between pre-rehabilitation and post-rehabilitation for each objective turning outcome. For each outcome, the ratio of the mean difference over minimum detectable change will be calculated. Ratios >1 will indicate that the mean difference due to rehabilitation was greater than normal variation between measurements, with larger ratios indicating larger effects due to rehabilitation.

### Refinement of Tasks and Outcomes for Future Clinical Use

To reduce the number of tasks and outcomes, a variable selection procedure will be implemented. First, objective turning outcomes for each task will be ranked according to their (1) AUC in differentiating people with mTBI from healthy control subjects, (2) correlation with functional performance in the civilian-relevant task, (3) correlation with functional performance in the military-relevant task, and (4) ratio of mean effect of rehabilitation over minimum detectable change. Outcome–task combinations will then be ordered according to the sum of their ranks across these four categories, where a lower total is better. The outcome, and associated task, with the lowest total sum will be identified and put forward in future applications and clinical training tools. Additionally, a civilian-specific task/outcome combination will be identified by summing the ranks, but excluding the rank corresponding to the correlation with functional performance in the military-relevant task. A military-specific task/outcome combination will be identified similarly, but summing all ranks except the correlation with functional performance during the civilian-specific task.

## Discussion

The functional deficits after mTBI can be subtle and are often overlooked in routine clinical assessments. Overt balance and mobility deficits during clinical tests typically resolve within 3 to 5 days after mTBI (83), but persistent, subtle abnormalities have been detected using sophisticated instrumentation and analysis techniques (53, 84–91). Increasing the difficulty or complexity of a task, such as adding a simultaneous cognitive DT, can also reveal subtle abnormalities; numerous studies describe the ability of DT measures to detect subtle, residual deficits from mTBI that may not be detected by traditional single domain assessment strategies (28, 49, 50, 92). Tests of physical function, such as the FGA and HiMAT, assess global performance during these complex tasks, but lack objectivity and nuanced measurement scales (12) that limit the tests' sensitivities (93, 94). However, recent advances in wearable technologies have enabled objective, sensitive assessments of subtle motor deficits during common clinical tests (8, 9, 95). By leveraging wearable sensors to quantify turning, an ecologically relevant and complex task, we hope to improve clinical decision-making after mTBI.

Clinical feasibility and ceiling effects in high-performing individuals are two main challenges in designing ecologically relevant assessments for RTD decisions. In tackling these challenges, we considered the following principles—the RTD assessment must be limited in time and equipment and easily administered; the clinically feasible assessment should, on its face, be ecologically relevant; performance on the assessment should be directly associated with performance in real-world tasks; and there should be no ceiling caused by the measurement technique (25). These principles led to the adaptation of three clinically feasible turning tasks that can each be administered with little additional training. While these tasks directly quantify the speed of turning, an ecologically relevant action performed hundreds of times per day (45), the tasks themselves are constrained based on clinical feasibility; they do not incorporate some of the complexities of the real world. Thus, the ability of outcomes from these turning tasks to predict performance on more specialized tasks (our CAT and SUP) is critical to the ecological relevance. Finally, while these turning tasks are rather simple compared to a simulated battle drill, wearable sensors facilitate continuous, objective outcome measures without an arbitrary ceiling due to the method of measurement.

If we find the DT turning performance can improve diagnostic accuracy of standard clinical assessments, is associated with functional performance in ecologically relevant tasks, and is responsive to rehabilitation, the results of this study will motivate rapid adoption of DT turning assessments in mTBI rehabilitation and RTD decisions. However, we anticipate that a single outcome measure may not satisfy all of these criteria. By instrumenting various body segments with wearable inertial sensors, we will simultaneously capture multiple outcome measures, each quantifying distinct aspects of movement (Table 1), for each task. Current clinical assessments of physical function lack this feature; they provide only one global score. However, clinical assessments with too many outcomes can become unguided. Our procedure to refine the selection of tasks and outcomes will rank outcomes based on their individual attributes (e.g., diagnostic accuracy, predictive capacity, sensitivity to change), as well as their overall combined attributes. These rankings will then guide the selection of tasks and outcomes that are ultimately recommended for clinical implementation.

Occupational demands and physical capabilities vary widely between and within civilians and military populations. Thus, adequate performance on the proposed turning tasks may be viewed as the bare minimum standard for RTD decisions. Because of the vast heterogeneity in occupational demands and physical capabilities both within and outside of the military, the current study is not designed to determine how performance varies by these factors. However, we anticipate addressing the extent to which performance differs between general civilian and military populations. If military populations perform differently (e.g., turn faster), it supports the use of population-adjusted normative ranges. Yet, even population adjusted normative ranges fail to address the variability within each subset due to occupation or physical health. Accordingly, the results of this study may be seen as a stepping stone for more individualized, and perhaps more complex, RTD assessments based on specific occupational duties.

Similar heterogeneity exists in the timeline of care and symptomology for individuals with mTBI. Based on recent evidence that active rehabilitation can be initiated within several weeks after mTBI and may yield greater gains than a traditional wait-and-see approach (96–99), the recent Clinical Practice Guidelines for Physical Therapy Evaluation and Treatment after mTBI state time since injury should not independently drive decisions about rehabilitative care (100). As a consequence, an ideal RTD assessment should be based on the occupational or situational demands one will face and should be applicable to individuals at various times since injury with diverse symptomology. This study will address whether the proposed tests are suitable as a universal RTD assessment, regardless of time since injury or symptom presentation. Yet, it is possible that performance will vary based on these factors. We will probe these relationships and adjust our planned analyses as appropriate pending the distributions of our final sample.

Because of the large sample size, multisite design, and inclusion of both civilian and active duty military populations, the results of this study are generalizable and can be rapidly implemented into clinical practice. To translate the results of this study into clinical environments, we will (1) develop standardized administration manuals and scoring sheets for each turning task/outcome selected for clinical use and (2) make recommendations to integrate these training and administration materials into DoD professional development and training courses for rehabilitation specialists. To facilitate widespread implementation, we will publish all analysis algorithms and clinical protocols on freely available, open-access repositories. Finally, we plan to seek future funds to translate the analysis algorithms and protocols to ubiquitous mobile technologies (e.g., cell phones) that do not rely on relatively expensive and specialized commercial technologies.

## Ethics Statement

The studies involving human participants were reviewed and approved by Oregon Health and Science University IRB, University of Utah IRB, Allina Health IRB, U.S. Army Medical Research and Materiel Command (USAMRMC), Office of Research Protections (ORP), Human Research Protection Office (HRPO). The patients/participants provided their written informed consent to participate in this study.

## Author Contributions

PF, MW, LD, ML, JA, AS, HR, and LK contributed to the study design and procurement of funding. CH and LP contributed to the implementation of the protocol across sites. PF wrote the first draft of the manuscript. All authors contributed to editing and revising the manuscript.

## Conflict of Interest

The authors declare that the research was conducted in the absence of any commercial or financial relationships that could be construed as a potential conflict of interest.
